# Gibberellin is not a regulator of *miR156* in rice juvenile-adult phase change

**DOI:** 10.1186/1939-8433-5-25

**Published:** 2012-09-22

**Authors:** Nobuhiro Tanaka

**Affiliations:** Graduate School of Agricultural and Life Sciences, University of Tokyo, Tokyo, 113-8657 Japan

**Keywords:** Gibberellin, *miR156*, *miR172*, Os*SPL* s

## Abstract

Plant hormone gibberellin (GA) promotes juvenile-adult phase change in higher plants. To confirm the functions of GA in rice, I used dwarf mutant *d18-dy*. *d18-dy* is a loss-of-function allele of *D18*, which encodes *GA3ox2*. *d18-dy* mutant exhibited long juvenile phase in morphological traits such as the size of the shoot apical meristem (SAM), shape of leaf blades, presence or absence of midribs and node–internode differentiation in stem. In contrast, expression patterns of juvenile-adult phase change markers *miR156* and *miR172* were similar between wild type and *d18-dy*. In addition, *d18-dy* mutation and GA did not affect expression levels of downstream genes of *miR156*. GA does not function upstream of *miR156* in juvenile-adult phase change.

**Electronic supplementary material:**

The online version of this article (doi:10.1186/1939-8433-5-25) contains supplementary material, which is available to authorized users.

## Background

Juvenile and adult phases are distinguished by several morphological markers (Lawson and Poethig 1995; Telfer et al. 1997; Asai et al. 2002). Juvenile-adult phase change is regulated by *miR156*, *miR172* and gibberellin (GA) in many higher plants (Lawson and poethig 1995; Telfer et al. 1997; Wu and Poethig 2006; Wang et al. 2011; Tanaka et al. 2011). However, the molecular mechanism involved in the juvenile-adult phase change is still unclear.

In *Arabidopsis*, juvenile leaves are round without abaxial trichomes, and adult leaves are long and serrated with abaxial trichomes (Telfer et al. 1997). In maize, leaves have epicuticular wax in juvenile phase, and adult leaves have no wax (Lawson and Poethig 1995). From these morphological markers, a lot of juvenile-adult phase change related mutants are reported in *Arabidopsis* and maize (Moose and Sisco 1996; Chuck et al. 2007; Schwarz et al. 2008; Smith et al. 2009). The observation of these mutants revealed that *miR156* had significant roles in juvenile-adult phase change (Wu and Poethig 2006; Chuck et al. 2007; Wu et al. 2009). In the early vegetative stage, transcription level of *miR156* exceeds that of *miR172*, whereas in later vegetative stage, the inverse pattern is seen (Wu and Poethig 2006; Chuck et al. 2007). In *Arabidopsis*, overexpression of *miR156* causes a prolonged juvenile phase (Wu and Poethig 2006). In addition, *miR156* overexpressed mutant, *Corngrass1* (*Cg1*) shows long juvenile phase in maize (Chuck et al. 2007). The *glossy15 (gl15*) mutant shortens the juvenile phase in the maize epidermis (Moose and Sisco 1996); *GL15* is an *AP2*-like gene that is the target of *miR172* (Lauter et al. 2005). In addition, *miR156* also controls juvenile-adult phase change in trees (Wang et al. 2011). *miR156* inhibits juvenile-adult phase change via repression of *SQUAMOSA PROMOTER BINDING PROTEIN-LIKE* (*SPL*) family genes resulting in decrease of *miR172* (Wu et al. 2009). Thus, *miR156* and *miR172* are key regulators in the juvenile–adult phase change.

Plant hormone Gibberellin (GA) is involved in the regulation of plant growth. GA is also known as adult phase promoter in *Arabidopsis* and maize (Lawson and Poethig 1995; Telfer et al. 1997; Telfer and Poethig 1998). GA deficient mutant, *ga1-3* exhibits dwarfism and glabrous leaf without abaxial trichome in *Arabidopsis* (Telfer et al. 1997). In maize *d1* and *d3* mutants, the expression of leaf epidermal wax is prolonged and the expression of leaf epidermal hairs is delayed compared with wild type (Lawson and Poethig 1995).

There are a few reports that examine the relationship between *miR156* and GA (Schwarz et al. 2008; Wang et al. 2009). Because the expression level of *SPL9* was similar in wild type and *ga1-3* mutant in *Arabidopsis* (Wang et al. 2009), *miR156* and GA related genes may function independently. However, the relationship between *miR156* and GA in juvenile-adult phase change is not confirmative.

In rice, a lot of GA-biosynthesis-deficient mutants are reported (Sasaki et al. 2002; Sakamoto et al. 2004). However no reports described how GA is related to juvenile-adult phase change in rice except Tanaka et al. (2011). Almost all molecular genetic studies of juvenile-adult phase change have been confined to *Arabidopsis* and maize. However, considerable number of morphological and physiological traits known to differ between the juvenile and adult phases are reported in rice, including the size of the shoot apical meristem (SAM), size and shape of leaf blades, presence of midribs, vascular orientation in the stem, node–internode differentiation, and photosynthetic rate (Itoh et al. 2005), thus rice is useful plant to understand the juvenile-adult phase change.

In this report, I examined *d18-dy* mutant from the stand point of juvenile-adult phase change. Expression patterns of *miR156* and *miR172* in *d18-dy* demonstrate that GA regulates juvenile-adult phase change independently of *miR156*-related pathway.

## Results

### Vegetative phenotypes of *d18-dy*

**Figure 1:**
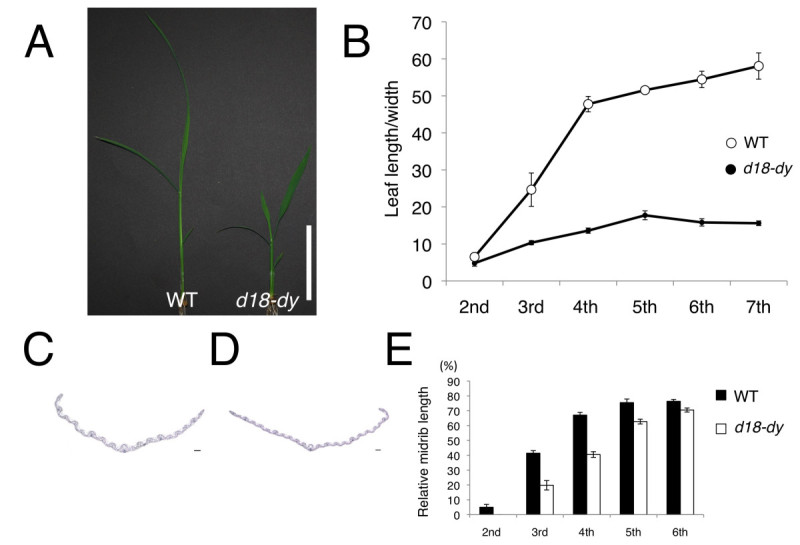
**Phenotypes of leaf in** ***d18-dy*** **plants.** (**A**) 2-week-old wild type (Taichung 65) and *d18-dy*. Bar = 5 cm. (**B**) Change in the ratio of leaf blade length to width in wild type and *d18-dy* (**C**) Cross section of *d18-dy* 3rd leaf blade cut at 25% from the base. (**D**) Cross section of *d18-dy* 4th leaf blade cut at 45% from the base. Bars = 100 μm. (**E**) Change of relative midrib length in leaf blades during development in wild type and *d18-dy.* Midrib length is shown as a percentage against total blade length. Data represent means ± SD in (**B**) and (**E**) (n = 5).

To confirm the function of GA as adult phase promoter, I examined the phenotypes of GA deficient mutant, *d18-dy*. Rice *D18* encodes *GA3ox2,* and the loss of function allele *d18-dy* causes severe dwarfism (Figure 1A). *d18-dy* has 9 bp deletion in the first exon of *GA3ox2* gene (Sakamoto et al. 2004). GA 3-oxidase oxidizes GA_20_ and GA_9_ to synthesize active GA, GA_1_ and GA_4_. The GA3ox deficient mutant is convenient to understand the function of GA in juvenile-adult phase change. First, I examined leaf shape that is defined as the ratio of leaf blade length to width. In wild type, the ratio drastically increased with elevation of leaf positions (Figure 1B). In *d18-dy*, even the ratio of the 7th leaf blade was below that of wild type 3rd leaf (Figure 1B). Second, I evaluated the presence of midrib in wild type and *d18-dy* leaf blades. The extent of midrib formation is a good marker for evaluating the juvenility in rice (Tanaka et al. 2011). I measured relative midrib length from the base of leaf blade. In *d18-dy* 3rd and 4th leaf blades, midrib covered approximately 20% and 40% of leaf blade, respectively (Figure 1C-E). These values were approximately half of those of wild type (Figure 1E). In both wild type and *d18-dy* 6th leaf, midrib covered more than 70% of leaf blade (Figure 1E). These results suggest that *d18-dy* shows prolonged juvenile phase, and it enters adult phase at around 6th leaf stage.

**Figure 2:**
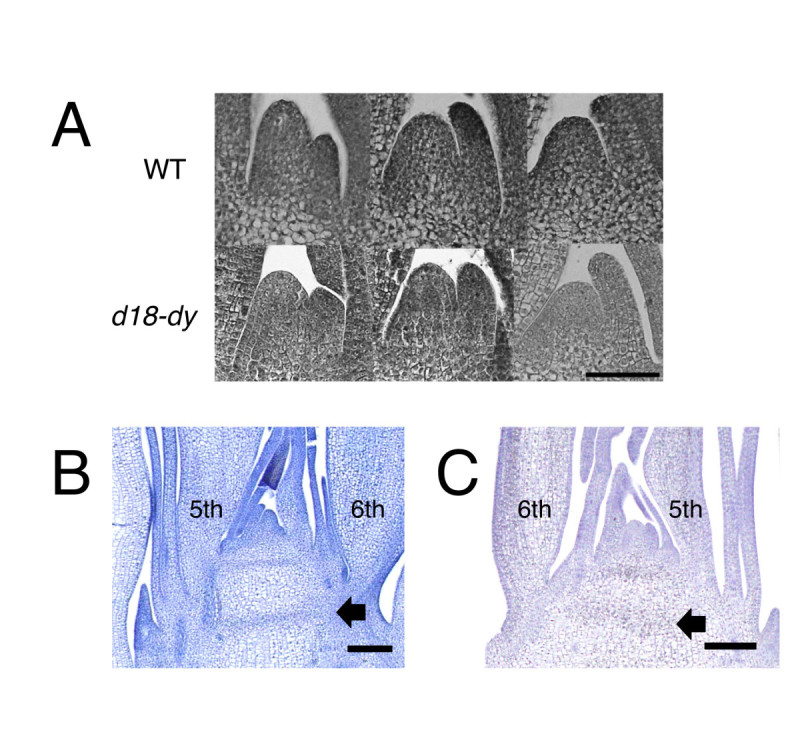
**Phenotypes of SAM and stem in** ***d18-dy*** **plants.** (**A**) Wild type and *d18-dy* shoot apices showing temporal change of SAM size. From left to right, 2nd leaf stage, 3rd leaf stage and 4th leaf stage. Bar = 50 μm. (**B**) Longitudinal section of 14-day-old wild type stem. (H) Longitudinal section of 20-day-old *d18-dy* stem. Arrows indicate leaf base nodes. Bars = 100 μm.

Next, I observed the changing pattern of SAM size during development. In *d18-dy*, SAM remained smaller than that of wild type till the 4th-leaf stage (Figure 2A). Additionally, *d18-dy* showed different node-internode differentiation. Above the 5th leaf, the stem has obvious node in wild type (Figure 2B). By contrast, node differentiation was not detectable until the insertion of 6th leaf in *d18-dy* (Figure 2C). These stem structures also indicate that *d18-dy* has long juvenile phase. From these morphological traits in *d18-dy*, I concluded that GA promoted juvenile-adult phase change in rice.

### GA and two miRNAs independently regulate juvenile-adult phase change

**Figure 3:**
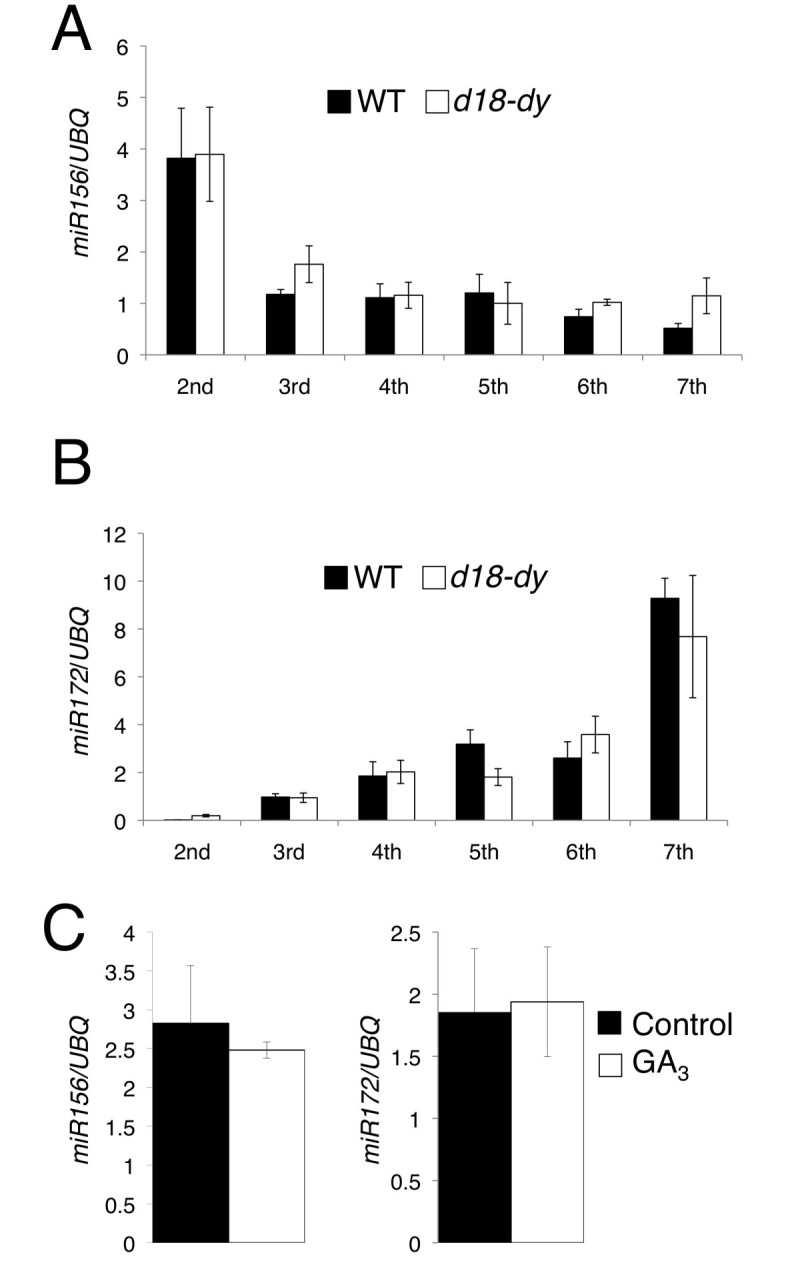
**Relationship between two miRNAs and GA.** (**A**) Expression of *miR156* in wild type and *d18-dy* 2nd to 7th leaves. (**B**) Expression of *miR172* in wild type and *d18-dy* 2nd to 7th leaves. (**C**) Expression of *miR156* and *miR172* in control and GA_3_ treated 3-day-old plants. Each value is the average of three independent real-time PCR assays. Data in (**A**), (**B**) and (**C**) represent means ± SD (n = 3).

To demonstrate the relationship between GA and two miRNAs in juvenile-adult phase change, I examined *miR156* and *miR172* expression patterns in wild type and *d18-dy* leaves (Figure 3A,B). In both wild type and *d18-dy* leaves*,* expression level of *miR156* was the highest in 2nd leaf, rapidly decreased to approximately one-third in 3rd leaf (Figure 3A), and was maintained at low level until the 7th leaf (Figure 3A). Wild type and *d18-dy* also showed similar expression pattern of *miR172*: the expression was quite low in 2nd leaf, and increased dramatically toward the 7th leaf (Figure 3B). In conclusion, expression patterns of the two miRNAs in *d18-dy* were identical to those in wild type (Figure 3A,B). From normal expression patterns of two miRNAs and retarded juvenile-adult phase change in *d18-dy*, I estimated that GA promotes adult phase transition independently of *miR156* and *miR172*. To confirm the relationship between GA and two miRNAs, I treated wild type plants with GA_3_. There is a report that the expression level of GA deactivation gene, *GA2ox4* is up-regulated by GA treatment (Yamaguchi 2008). In GA treated plants, expression level of *GA2ox4* was higher than in control plants (Additional file 1: Figure S1). This indicates that the experimental system of GA treatment is normally functioning. Subsequently I examined *miR156* and *miR172* expression levels in control and GA_3_ treated plants. Both expression levels of two miRNAs were not affected by the application of GA_3_ (Figure 3C). Thus I concluded that GA did not regulate the onset of adult phase upstream of *miR156*.

### Expression patterns of Os*SPL*

**Figure 4:**
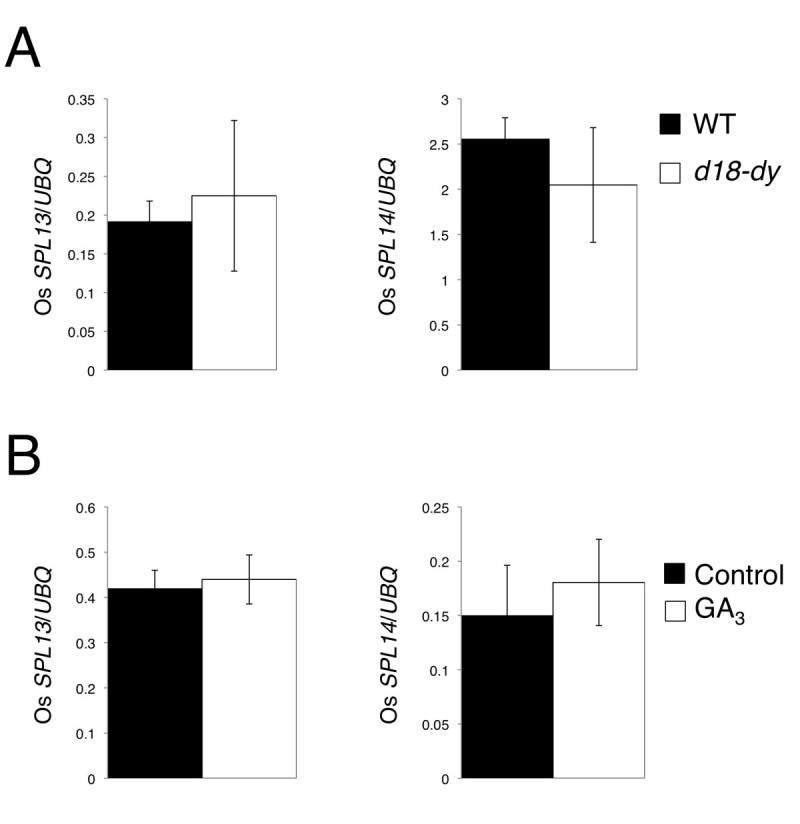
**Expression patterns of Os** ***SPL*** **s in** ***d18-dy*** **.** (**A**) Expression of Os *SPL13* and Os *SPL14* in wild type and *d18-dy* 2nd leaves. (**B**) Expression of Os *SPL13* and Os *SPL14* in control and GA_3_ treated 3-day-old plants. Each value is the average of three independent real-time PCR assays. Data in (**A**), (**B**) and (**C**) represent means ± SD (n = 3).

To further confirm the hypothesis that GA functions independently of *miR156* pathway in juvenile-adult phase change, I examined the expression levels of Os*SPL13* and Os*SPL14* that are the ortholog of *Arabidopsis SPL3* and *SPL9* (Xie et al. 2006). Both Os*SPL13* and Os*SPL14* contain *miR156* target sites, and *OsSPL14* ortholog, *SPL9* regulates *miR172* expression positively in *Arabidopsis* (Wu et al. 2009). The expression levels of both Os*SPL13* and Os*SPL14* in leaves were almost comparable between wild type and *d18-dy* (Figure 4A). Next, I examined Os*SPL13* and Os*SPL14* expression in GA_3_ treated plants. Both Os*SPL13* and Os*SPL14* expression levels were similar between control and GA_3_ treated plants (Figure 4B). These results indicate that GA does not affect the expression of *miR156-* target genes. I again confirmed that GA-related pathway did not act upstream of *miR156*.

## Discussion

### GA and *miR156* function independently to regulate juvenile-adult phase change

The study of juvenile-adult phase change is difficult because it is accompanied by subtle morphological traits. In addition, the morphological markers for juvenile-adult phase change are different among plant species. However, *miR156* and GA are reported as common juvenile-adult phase change regulator in many flowering plants (Lawson and Poethig 1995; Telfer et al. 1997; Wu et al. 2009; Chuck et al. 2007; Wang et al. 2011; Tanaka et al. 2011). GA-related mutant, *d18-dy* showed prolonged juvenile phase, such as delayed midrib formation, small leaf size, small SAM size and delayed node-internode differentiation. These results strongly indicate that GA also promotes adult phase transition in rice.

The expression patterns of *miR156* and *miR172* were comparable between wild type and *d18-dy* plants. These results suggest that GA and *miR156* regulate juvenile-adult phase change through independent genetic pathway. Over-expressed *MIR156* line in *Arabidopsis* shows prolonged juvenile phase, however it can enter the reproductive phase (Schwarz et al. 2008). In rice, *MIR156* over-expressed plant also showed dwarfism, but developed to flowering stage (Xie et al. 2012). Similarly, GA-deficient mutants can enter the reproductive phase in many flowering plants (Telfer et al. 1997; Itoh et al. 2004). These phenotypes indicate that *miR156* functions redundantly with GA in the determination of the exact time of juvenile-adult phase change. Double mutant of *MIR156* over-expressed plant and GA deficient mutant might show persistent juvenile phase.

In *d18-dy* 6th leaf stage, the plant was obviously dwarf. This indicates that amount of active GA in *d18-dy* is still low at 6th leaf stage. However, *d18-dy* showed normal midrib formation at this stage (Figure 1E). This suggests that the function of GA is less important for midrib formation after 6th leaf stage than during 2nd-to-5th leaf stages.

Short plastochron is also known as juvenile phase character in rice (Itoh et al. 2005). *MIR156* over-expressed plant had more leaves than wild type (Xie et al. 2012). In contrast, the rate of leaf initiation was comparable between *d18-dy* and wild type. These indicate that *miR156* and GA have different functions in regulation of plastochron.

## Conclusions

Long juvenile phase phenotype of *d18-dy* indicated that GA is the adult phase promoter in rice. In higher plants, *miR156* and *miR172* are also known as juvenile-adult phase change regulator. Our study demonstrated that GA does not regulate juvenile-adult phase change via a pathway of *miR156*. Moreover, GA does not regulate Os*SPL* s that are the *miR156*-target genes.

## Methods

### Plant materials

I used *d18-dy* that is a dwarf mutant defective in GA biosynthestic gene encoding GA3 OXIDASE 2. Mutants and wild type plants were grown in pots under natural field conditions.

### Paraffin sectioning

Leaves and shoot apices were fixed with FAA (formalin:acetic acid:50% ethanol, 1:1:18) for 24 h at 4°C. They were dehydrated in a graded ethanol series and embedded in Paraplast plus (McCormick Scientific). Microtome sections (8 μm thick) were stained with Delafield's hematoxylin.

### Gene expression profiling

The real-time PCR for *miR156* and *miR172* was performed using TaqMan MicroRNA Assay (Applied Biosystems). Total RNA was extracted using TRIzol reagent (Invitrogen) from wild type 2nd, 3rd, 4th, 5th, 6th and 7th leaves. In addition, RNA was also isolated from *d18-dy* 2nd, 3rd, 4th, 5th, 6th and 7th leaves. To quantify the *miR156* and *miR172* expression, PCR was performed using the TaqMan Fast Universal PCR Master Mix (Applied Biosystem). I used *UBQUITIN* (*UBQ*) as inner control and qPCR was conducted using SYBR green master mix (Applied Biosystem). For quantifying the *UBQ* expression, Real-time PCR was performed using High Capacity RNA-to-cDNA Master Mix (Applied Biosystems).

To observe the Os *SPL13 and* Os *SPL14* expressions, total RNA was isolated from wild type and *d18-dy* 2nd leaves. I used *UBQ* as inner control and qPCR was conducted using SYBR green master mix (Applied Biosystem). Real-time PCR was performed using High Capacity RNA-to-cDNA Master Mix (Applied Biosystems).

For observing *miR156*, *miR172,* Os *SPL* s and *GA2ox4* expression patterns in GA treated plants, sterilized seeds of wild type were plated on MS medium (Murashige and Skoog 1962) containing 10^-5^ M GA_3_ (SIGMA). Plants were grown in a growth chamber under the continuous light at 28°C. Total RNA was extracted using TRIzol reagent (Invitrogen) from 3-day-old wild type seedlings. To quantify the *miR156* and *miR172* expression, PCR was performed using the TaqMan Fast Universal PCR Master Mix (Applied Biosystem). I used *UBQUITIN* (*UBQ*) as inner control and qPCR was conducted using SYBR green master mix (Applied Biosystem). For quantifying the Os *SPL13*, Os *SPL14* and *UBQ* expression, Real-time PCR was performed using High Capacity RNA-to-cDNA Master Mix (Applied Biosystems). PCR was performed using the StepOnePlus real-time PCR system (Applied Biosystems). For quantifying the *GA2ox4* and *UBQ* expression, Real-time PCR was performed using PrimeScript RT Master Mix (Takara). qPCR was conducted using SYBR Premix Ex Taq ll (Takara). PCR was performed using Thermal Cycler Dice TP800 (Takara). Gene-specific primes for Os *SPL13*, Os *SPL14 GA2ox4* and *UBQ* are listed in Additional file 2: Table S1 online.

## Electronic supplementary material

Additional file 1:**Figure S1.** Expression patterns of *GA2ox4* in GA_3_ treated plants. Expression of *GA2ox4* in control and GA_3_ treated 3-day-old plants. Each value is the average of three independent real-time PCR assays. Data represent means ± SD (n = 3). (PDF 76 KB)

Additional file 2:**Table S2.** List of primers for semi-quantitative RT-PCR. (DOC 26 KB)

Below are the links to the authors’ original submitted files for images.

Authors’ original file for figure 1

Authors’ original file for figure 2

Authors’ original file for figure 3

Authors’ original file for figure 4

Authors’ original file for figure 5
