# Supplementary figures and images for: Gibberellin is not a regulator of miR156 in rice juvenile-adult phase change
Source: Rice (N Y). 2012 Sep 22;5:25. doi: 10.1186/1939-8433-5-25 (PMC4883733; doi:10.1186/1939-8433-5-25)

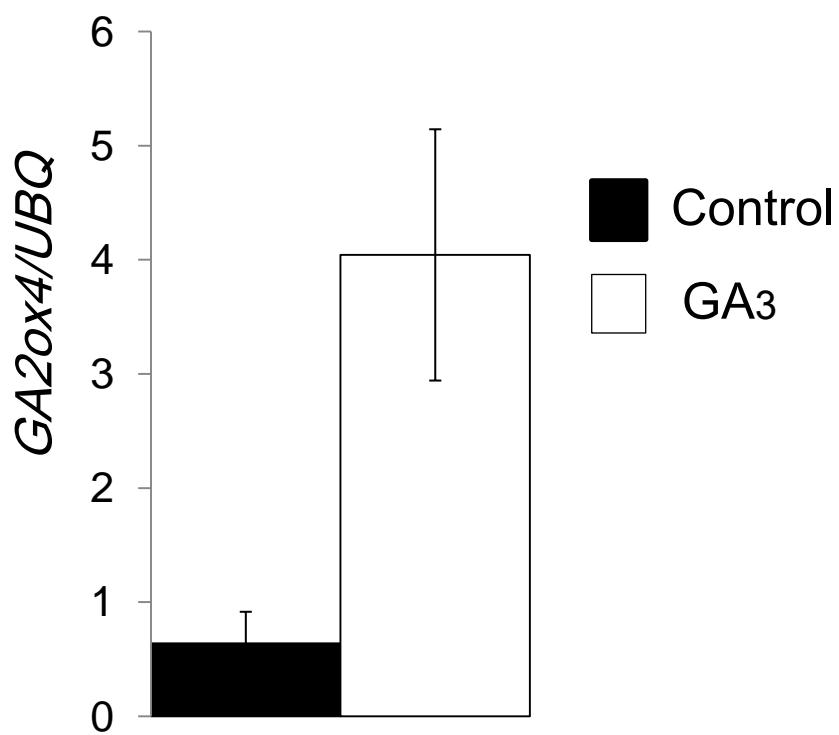

Supplement: Supplementary file 1 — Additional file 1:Figure S1. Expression patterns of GA2ox4 in GA3 treated plants. Expression of GA2ox4 in control and GA3 treated 3-day-old plants. Each value is the average of three independent real-time PCR assays. Data represent means ± SD (n = 3). (PDF 76 KB) [file 12284_2012_24_MOESM1_ESM.pdf]

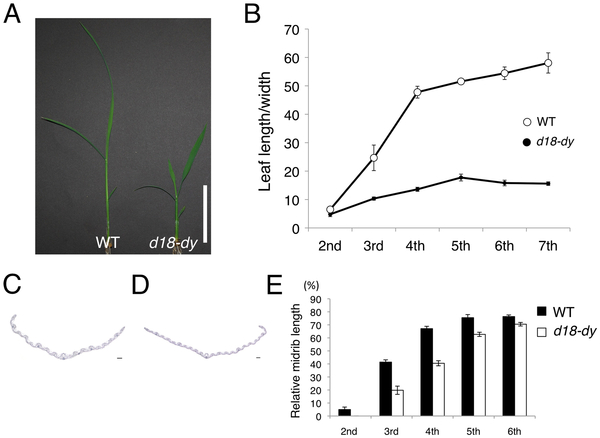

Supplement: Supplementary file 3 — Authors’ original file for figure 1 [file 12284_2012_24_MOESM3_ESM.jpeg]

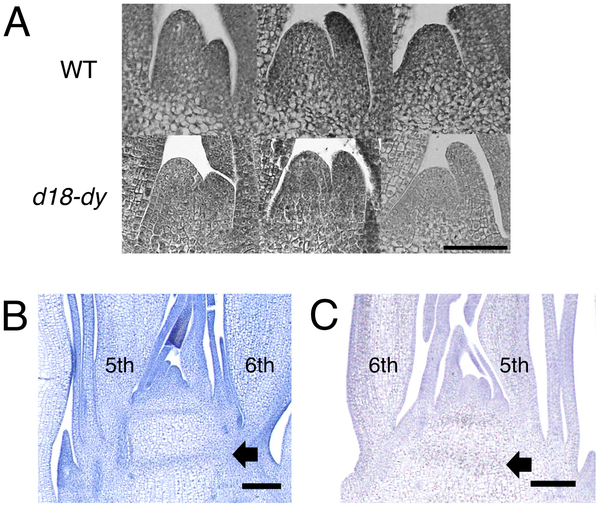

Supplement: Supplementary file 4 — Authors’ original file for figure 2 [file 12284_2012_24_MOESM4_ESM.jpeg]

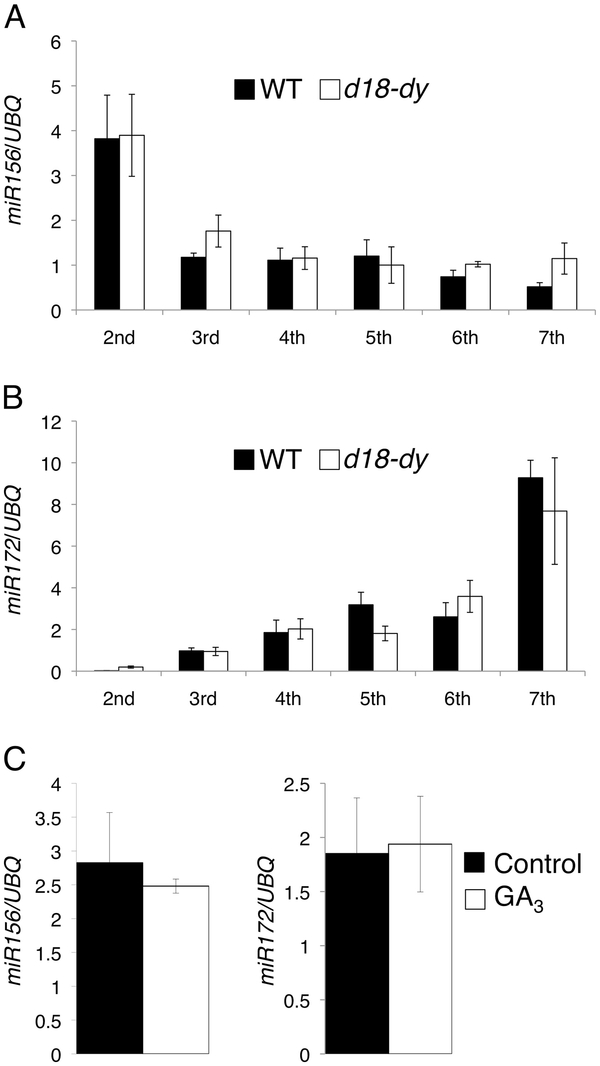

Supplement: Supplementary file 5 — Authors’ original file for figure 3 [file 12284_2012_24_MOESM5_ESM.jpeg]

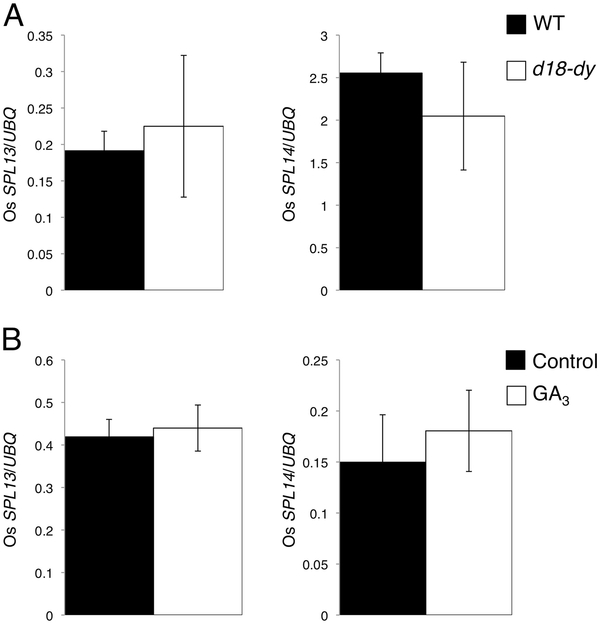

Supplement: Supplementary file 6 — Authors’ original file for figure 4 [file 12284_2012_24_MOESM6_ESM.jpeg]
