# Supplementary material for: Gibberellin is not a regulator of miR156 in rice juvenile-adult phase change
Source: Rice (N Y). 2012 Sep 22;5:25. doi: 10.1186/1939-8433-5-25 (PMC4883733; doi:10.1186/1939-8433-5-25)
Supplement: Supplementary file 2 — Additional file 2:Table S2. List of primers for semi-quantitative RT-PCR. (DOC 26 KB) [file 12284_2012_24_MOESM2_ESM.doc]

**Supplemental Data**

Table S1. List of primers for semi-quantitative RT-PCR

| Gene name Primers |
| --- |
| Os *SPL13* 5’-CGCCGTTCCAGATCAGATAA-3’  5’-TAGTGGCACGAACACACACA-3’  Os *SPL14* 5’-TAGCCATCATGCCCACTTCT-3’  5’-TCTTCAGTTCCATGACTCGC-3’  *GA2ox4* 5’-TTGCAGGTTCTGACCAATGG-3’  5’-AATGGTGCAATCCTCTGTGCTA-3’  *UBQ* 5’-ACCACTTCGACCGCCACTACT-3’  5’-ACGCCTAAGCCTGCTGGTT-3’ |
